# Comparison of bone marrow, disseminated tumour cells and blood-circulating tumour cells in breast cancer patients after primary treatment

**DOI:** 10.1038/sj.bjc.6604773

**Published:** 2008-11-25

**Authors:** M J Slade, R Payne, S Riethdorf, B Ward, S A A Zaidi, J Stebbing, C Palmieri, H D Sinnett, E Kulinskaya, T Pitfield, R T McCormack, K Pantel, R C Coombes

**Affiliations:** 1 Department of Oncology, Imperial College, MRC Cyclotron Building, London W12 0NN, UK; 2 Institute of Tumor Biology, University Medical Center Hamburg-Eppendorf, Hamburg D-20246, Germany; 3 Department of Medical Oncology, Imperial College Healthcare NHS Trust, Charing Cross Hospital, Fulham Palace Road, London W6 8RP, UK; 4 Statistical Advisory Service, Imperial College, South Kensington Campus, London SW7 1NA, UK; 5 Veridex LLC, 50-100 Holmers Farm Way, High Wycombe, HP12 4DP, UK; 6 Veridex LLC, 33 Technology Drive, Warren, NJ 07059, USA

**Keywords:** micrometastasis, detection, disseminated, circulating, CellSearch

## Abstract

The purpose of this study was to determine whether primary breast cancer patients showed evidence of circulating tumour cells (CTCs) during follow-up as an alternative to monitoring disseminated bone marrow tumour cells (DTCs) by immunocytochemistry and reverse transcriptase (RT)–PCR for the detection of micrometastases. We planned to compare CTC and DTC frequency in low-risk and high-risk patients. We identified two cohorts of primary breast cancer patients who were at low (group II, T_1_N_0_, *n*=18) or high (group III, >3 nodes positive (with one exception, a patient with two positive nodes) *n*=33) risk of relapse who were being followed up after primary treatment. We tested each cohort for CTCs using the CellSearch system on 1–7 occasions and for DTCs by immunocytochemistry and RT–PCR on 1–2 occasions over a period of 2 years. We also examined patients with confirmed metastatic disease (group IV, *n*=12) and 21 control healthy volunteers for CTCs (group I). All group I samples were negative for CTCs. In contrast, 7 out of 18 (39%) group II primary patients and 23 out of 33 (70%) group III patients were positive for CTCs (*P*=0.042). If we count only samples with >1 cell as positive: 2 out of 18 (11%) group II patients were positive compared with 10 out of 33 (30%) in group III (*P*=0.174). In the case of DTCs, 1 out of 13 (8%) group II patients were positive compared with 19 out of 27 (70%) in group III (*P*<0.001). Only 10 out of 33 (30%) patients in group III showed no evidence of CTCs in all tests over the period of testing, compared with 11 out of 18 (61%) in group II (*P*=0.033). A significant proportion of poor prognosis primary breast cancer patients (group III) have evidence of CTCs on follow-up. Many also have evidence of DTCs, which are more often found in patients who were lymph node positive. As repeat sampling of peripheral blood is more acceptable to patients, the measurement of CTCs warrants further investigation because it enables blood samples to be taken more frequently, thus possibly enabling clinicians to have prior warning of impending overt metastatic disease.

Breast cancer is the most common cancer among women in the United States and Europe. Although mammographic screening and the judicious use of adjuvant systemic therapy have improved the survival from this disease, many patients still relapse and develop metastatic disease. Metastases inevitably result in the death of the patient.

Over the past 10 years, long-term follow-up of patients in trials designed to evaluate adjuvant endocrine or cytotoxic chemotherapy has indicated that cure can be achieved in a proportion of patients by these treatments. For this reason, considerable efforts have been made to discover a means of monitoring these patients, in the hope of finding a test that would distinguish those who need further sequential adjuvant therapy from those for whom this treatment would not be necessary. This has become particularly important on account of our recent studies showing that ‘interval’ or ‘switching’ techniques improve disease-free (Coombes *et al*, 2004) and overall survival (Coombes *et al*, 2007).

Several studies have now shown that the presence of occult metastases in the bone marrow identifies a population of patients at high risk for recurrence (Redding *et al*, 1983; Cote *et al*, 1988; Diel *et al*, 1996; Mansi *et al*, 1999; Braun *et al*, 2000c; Wiedswang *et al*, 2003; Naume *et al*, 2004). In our original study (Neville *et al*, 1983), the presence of bone marrow occult metastases was correlated with tumour stage and vascular invasion, both of which are known predictors of poor prognosis. Other studies of note are Diel *et al* (1996) and Braun *et al* (2000b) who analysed bone marrows from 727 and 552 patients, respectively, using immunocytochemistry (ICC). Both these studies have shown that the occult bone marrow metastases were associated with larger tumour size, lymph mode involvement and high-grade tumour. The presence of micrometastases in bone marrow at surgery has been shown to be an independent prognostic factor in 817 breast cancer patients (Wiedswang *et al*, 2003). In a pooled analysis of 4703 patients with stages I, II or III breast cancer with 10-year follow-up (Braun *et al*, 2005), micrometastases at diagnosis were detected in 30.6% of patients. Those patients with micrometastases had larger tumours and higher histological grade and more frequent lymph node metastases and hormone receptor-negative tumours. This study shows that the presence of micrometastases in the bone marrow at the time of diagnosis of breast cancer was an independent predictor of a poor outcome and is associated with poor prognosis.

In studies from our laboratory and from others, it has been shown that it is possible to detect residual disseminated bone marrow tumour cells (DTCs) in the bone marrow, and circulating tumour cells (CTCs) in the peripheral blood during follow-up. However, the number of cells in the bone marrow is small even after using the best available techniques (i.e., using a pancytokeratin antibody (A45-B/B3), which is well characterized for DTC studies (Braun *et al*, 2000b)) for staining 2 × 10^6^ cells and analysing automatically. On account of the difficulties inherent in subjecting patients to regular bone marrows, as well as the small number of cells detected in primary patients with no evidence of overt metastases, such tests have been difficult to apply in routine clinical practice. Furthermore, until recently, the peripheral blood has been shown to be generally negative for CTCs in all but a minority of patients, with most series showing 1–5% of patients found to be positive (Schoenfeld *et al*, 1997; Slade *et al*, 2005).

Many groups have attempted to develop a reproducible methodology for CTC detection to improve the detection rate. One of these is the CellSearch™ (Veridex LLC, Warren, NJ, USA) system with which Cristofanilli *et al* (2004) carried out a study of 177 patients with metastatic breast cancer. The results from this approach indicated that approximately 70% of patients with metastatic breast cancer have >1 CTC/7.5 ml of peripheral blood. They also found that patients with five or more CTCs per 7.5 ml of blood had a significantly worse progression-free and overall survival than patients with <5 cells per 7.5 ml of blood (progression-free survival was 2.1 *vs* 7.0 months and overall survival was 8.2 *vs* 18 months). It has subsequently been shown (Hayes *et al*, 2006) in this group of patients that elevated CTCs at any time during therapy are an accurate indicator of rapid disease progression and mortality.

We therefore decided to perform a pilot study to determine whether monitoring for CTCs using the CellSearch system was comparable with our current micrometastatic monitoring system using ICC and reverse transcriptase (RT)–PCR of bone marrow samples. This was performed in a group of patients on follow-up after a diagnosis of breast cancer, and none of them had evidence of overt metastases.

## Materials and methods

### Patients

The study was approved by each local ethics committee and conducted in accordance with the declaration of Helsinki and all patients gave written informed consent. Samples were blinded for analysis and patients understood that the results would not be made available to them. Patients diagnosed with early stage breast cancer, who were on routine follow-up, were invited to take part in the study. All women were attending local hospitals in the West London Cancer Network. All patients had previously histologically confirmed primary breast cancer and no evidence of distant metastases on chest radiology, bone scanning or liver ultrasound. It was not possible to compare the two sampling sites (i.e., blood and bone marrow), as the samples were not taken at identical time points for ethical reasons.

### Breast cancer patients

To have a group of patients who were at very low risk of having recurrence in the future, we decided to take the subset of patients from our published study (Slade *et al*, 2005) to include only those patients with T_1_N_0_ (i.e., those who had a tumour ⩽2 cm)and who were node negative (group II) 4–13 years post-surgery. For the high-risk group (group III), we included patients who previously had >3 node-positive breast cancer (with one exception, a patient with two positive nodes) and were on follow-up and who had no sign or symptom of recurrence. We examined 3 × 7.5 ml samples of blood from all of these patients together with the same amount of blood from a cohort of women with no past history of breast cancer (negative control – group I). We repeated the sampling procedure 1–7 times over a 2-year period. As positive controls, we took 1 × 7.5 ml blood from a group of 12 patients with overt metastatic breast cancer (group IV).

### Preparation of bone marrow samples

The skin was incised before the aspirates were taken to minimise the risk of epithelial contamination. Between 2 and 5 ml of bone marrow was aspirated into syringes primed with preservative-free heparin (Leo Labs, Risborough, UK). Bone marrow samples were prepared as described earlier (Slade *et al*, 1999; Smith *et al*, 2000). Ficoll (GE Healthcare, Chalfont St Giles, UK) was used to separate the mononucleocytes; these were counted and aliquoted for ICC on the basis of ideally 6 × 10^6^ cells for each methodology but with a minimum of 3 × 10^6^ cells for each. Those undertaking the ICC were blind to the clinical status and identity of the patients and their earlier assay results.

### Immunocytochemistry

Cells were cytocentrifuged at a concentration of 5 × 10^5^ per cytospin, air dried and stained as described earlier (Pantel *et al*, 1994; Slade *et al*, 1999; Smith *et al*, 2000). A total of six areas were stained, of which four were stained for the presence of cytokeratin-positive cells and two were isotype controls. The primary antibody (A45-B/B3 Micromet, Munich, Germany) directed to a common epitope of cytokeratin (Stigbrand *et al*, 1998) was used at 2 *μ*g ml^−1^. The rabbit anti-mouse antiserum (Z259; Dako, Hamburg, Germany) and the alkaline phosphatase anti-alkaline phosphatase (APAAP) complex (D651; Dako) were used as recommended by the manufacturer and the reaction developed with new fuchsin. An isotype IgG1 mouse myeloma antibody MOPC-21 (Sigma Aldrich, Poole, UK) served as negative control and the MCF-7 cell line as a positive control (Pantel *et al*, 1994; Slade *et al*, 1999; Smith *et al*, 2000). The cytospins were counterstained with haematoxylin and screened using the Automated Cellular Imaging System (ACIS) (Carl Zeiss Ltd, Welwyn Garden City, UK). Samples that were isotype positive were deemed uninterpretable and therefore excluded from the results.

### CellSearch

Blood, 7.5 ml from the metastatic patients and 3 × 7.5 ml from the control and primary breast cancer patients, was collected in CellSave™ preservative tubes (Immunicon, Huntingdon, PA, USA) from patients in London, anonymised, and transported at room temperature to either the Institute of Tumor Biology in Hamburg or the Department of Oncology, Imperial College London for processing within 72 h of collection as recommended by the manufacturer. The CellSearch system was used for the isolation and enumeration of CTCs from each 7.5 ml of blood separately. The CellSearch Epithelial Cell Kit (Veridex) enriches the sample for cells expressing the epithelial-cell adhesion molecule (Ep-CAM) with antibody-coated magnetic beads, and labels the cells with the fluorescent nucleic acid dye 4,2-diamidino-2-phenylindole dihydrochloride (DAPI). Fluorescently-labelled monoclonal antibodies raised against leukocytes (CD45-allophycocyanin) and epithelial cells (cytokeratin 8, 18, 19-phycoerythrin) are used to distinguish epithelial cells from leukocytes. The identification and enumeration of CTCs were performed with the use of the CellTracks Analyzer (Immunicon), a semi-automated fluorescence-based microscopy system that permits computer-generated reconstruction of cellular images. Circulating tumour cells were defined as nucleated cells lacking CD45 and expressing cytokeratin in accordance with the criteria specified by Veridex.

### Real-time QPCR

Real-time QPCR for CK-19 and ABL was performed using the LightCycler™ (Roche Diagnostics, Mahnheim, Germany). The assay used the LightCycler DNA FastStart SYBR Green 1 kit (Roche Diagnostics) using 2 *μ*l of combined dNTPs, *Taq* DNA polymerase and SYBR Green plus 2.5 *μ*l of cDNA and 0.625 *μ*M of primers in a total volume of 20 *μ*l.

### Primers

CK-19S: 5′-GCGGGCAACGAGAAGCTAA-3′
CK-19Do: 5′-CTCATGCGCAGAGCCTGTT-3′
A2N: 5′-CCCAACCTTTTCGTTGCACTGT-3′
A4-: 5′-CGGCTCTCGGAGGAGACGTAGA-3′

The standard used for quantitation was the artificial construct described earlier (Slade *et al*, 1999), in the range 10^1^–10^4^ for CK-19 and 10^3^–10^6^ for ABL per 2.5 *μ*l.

### Statistical methods

Owing to the small numbers, exact two-sided non-parametric tests were used throughout at 5% level of significance. The exact Pearson *χ*^2^-test was used for testing association of two categorical variables, the Mann–Whitney test for the comparison of continuous variables between the two groups, and the Spearman correlation for correlations of two continuous variables.

## Results

To perform this pilot study, we recruited 51 primary breast cancer patients (18 group II T_1_N_0_ ‘low risk’ and 33 group III >3 nodes positive (with one exception, a patient with two positive nodes) ‘high risk’ patients) who were on routine follow-up, with no clinical evidence of disease, 4–13 years after diagnosis. No patient had any sign or symptom of metastatic breast cancer. Two control populations were also recruited, 21 non-cancer controls (group I) and 12 metastatic patients (group IV), to confirm earlier results with the CellSearch system (Cristofanilli *et al*, 2004, 2005; Riethdorf *et al*, 2007). Overall, 14 patients in group III and one patient in group II were taking adjuvant endocrine therapy. No patient changed treatment during the course of the monitoring period. Patients in group II had one bone marrow sample taken yearly. The higher risk patients in group III had two bone marrows taken 6–12 months apart. All patients had between 1–7 samples of peripheral blood taken (three tubes or 22.5 ml of blood at each time point) for immunocytochemical evaluation using the CellSearch system approximately 6–34 months apart. These patients also had an isotopic bone scan, liver ultrasonography and chest radiology, as well as blood evaluation for full haematological and biochemical screen to exclude overt metastatic disease. All but two patients had no evidence of metastatic disease. For ethical reasons, the non-cancer controls had two blood tests (15 ml) for the CellSearch system and only patient groups II and III had bone marrow aspirates taken. The number of CTCs and DTCs present in blood and bone marrow, respectively, were compared in the low- and high-risk primary breast cancer patient groups.

### CTC results

In the patients with overt metastases, we found CTCs in seven patients; the mean number of cells detected was 51 with a range of 0–301. Fewer patients (7 out of 18 (39%)) in group II compared with group III (23 out of 33 (70%)) had at least one CTC (*P*=0.042, exact *χ*^2^-test). Results are shown for all patients in Tables 1 and 2. We then analysed our results taking >1 cell as positive: 2 out of 18 (11%) patients in group II *vs* 10 out of 33 (30%) in group III were positive using this cutoff point (*P*=0.174, exact *χ*^2^-test).

Groups II and III patients were tested for CTCs using the CellSearch system at 1–7 time points. There was no difference in the number of tests between the groups (*P*=0.812), although the time from diagnosis to the start of testing and the time of intervals between tests were not associated with the results of the CTC tests within the groups (*P*-values 0.274 and 0.602). Neither oestrogen receptor (ER) or progesterone receptor (PR) status nor tumour size affected the proportion of the CTC-positive tests within each group (*P*=0.374 and *P*=0.238).

None of the non-cancer controls had any evidence of CTCs. Of the 18 group II patients five (28%) were positive at one time point, and two (11%) at two time points. By contrast, in group III, 13 (39%) patients were positive at one time point, six (18%) at two time points and four (12%) at three time points.

In all, only 10 out of 33 (30%) patients in group III showed no evidence of CTCs in all tests over the period of testing, compared with 11 out of 18 (61%) in group II (*P*=0.033). The negative patients may reflect the potentially small number of patients who will not clinically relapse. Two patients developed evidence of overt metastases during the course of the study. Both of these patients had positive results on the CellSearch system, and in each patient, DTCs were detected by ICC in one of the two bone marrows and by QPCR in two of the bone marrows.

The chances of finding positive results did not seem affected by whether or not the patients were receiving endocrine therapy at the time of sampling (*P*=0.389 and *P*=0.693 for groups II and III, respectively).

Over this period of sampling for CTCs, we obtained 68 bone marrow samples for DTC analysis; 13 patients in group II and one patient in group III were tested once, and 27 patients in group III at two time points. Overall, 1 out of 13 patients (8%) in group II were positive for DTCs in contrast to the 19 out of 27 positive at least once when tested at two time points in group III (*P*<0.001). Only 8 out of 27 (30%) patients in group III were negative at both time points, 16 out of 27 were positive once, and 3 out of 27 positive at two time points, but none had two or more cells at both time points (see Tables 1 and 2).

For bone marrow QPCR, none of the 13 patients in group II were positive in contrast to 16 of 27 patients tested at two time points in group III (*P*<0.001); 16 were positive at both time points.

We also studied the relationship between the DTC results and the CTC results. Patients consistently negative for DTCs in bone marrow (measured by ICC) can have positive CTCs (5 out of 12 in group II and 4 out of 8 in group III), but among the patients with positive DTCs, there is a high proportion of patients with positive CTCs (16 out of 19 (84%) in group III). These numbers were too small to reach statistical significance (*P*=0.145).

## Discussion

These data indicate that some patients who have had a diagnosis of breast cancer have evidence of micrometastatic breast cancer during follow-up, despite having no clinical evidence of metastases. Seven of 18 (39%) of the T_1_N_0_, group II patients were found to have CTCs compared with 23 of 33 (70%) of the patients with lymph-node-positive disease. Although the numbers of patients in this pilot study are small, the results suggest that the likelihood of finding CTCs and DTCs on follow-up is greater in the patients who are at higher risk of recurrence (*P*=0.042 and *P*<0.001, respectively) and that the numbers of CTCs are greater in the poor-risk patients.

We have previously published the fact that there is occasional evidence of DTCs on follow-up of patients over 4 years after treatment for primary breast cancer (Slade *et al*, 2005). Most patients show considerable variability. In this pilot study, we have intensively investigated a cohort of patients and found, on the basis of one or two separate tests over a 2-year follow-up period, that many patients show positive tests, sometimes confirmed by the CellSearch test for CTCs. It may be significant that a proportion of patients with bad prognosis on the basis of their tumour histology showed consistently negative results. None of these relapsed with overt disease during the course of the study, compared with two patients in the group with positive results.

There have been few studies in primary breast cancer patients, either at presentation or at follow-up. Wulfing *et al* (2006) discovered that 50% of patients had evidence of HER-2-positive cells in the blood and that this correlated with survival. It can be noted that some patients who were initially judged to have HER-2-negative tumours had HER-2-positive CTCs. It can be noted that some patients who were initially judged to have HER-2 negative tumours had HER-2 positive CTCs. A study of 456 primary patients showed that 28% had ⩾1 CTC in 3 × 7.5ml of blood but the presence of CTCs did not correlate with any prognostic features of the primary tumour (Rack *et al*, 2006). Studies such as this are necessary in order to determine a cut-off for positivity in primary patients (both this study and ours used a cut-off of a single CTC) with clinical follow-up and also studies in patients with benign breast disease.

Discrepancies between the findings for DTCs and CTCs may be explained either by differences in the methodology of detection (RT–PCR for CK-19 only, as compared with ICC for CK8, 18 and 19 for the DTCs and Ep-CAM enrichment, followed by pan-cytokeratin staining for CTCs), or because DTCs and CTCs represent two different levels of risk. It has been shown that CTCs have a half-life of 1–2.4 h (Meng *et al*, 2004) and are non-replicating (Muller *et al*, 2005) and that these must be replenished by replicating cells from elsewhere. Potentially, this could be the bone marrow; however, the DTCs, when in the bone marrow, are also non-replicating in the majority of primary breast cancer patients (Pantel *et al*, 1993; Muller *et al*, 2005). In each case, we have used the best available methodologies for detecting the cells and further studies on a larger cohort of patients along with improvements in assays, for example, CellSearch applied to bone marrow aspirates, may improve the correlation between CTCs and DTCs. With regard to the CTCs and DTCs being biologically and genetically different cells or DTCs in bone marrow being a subset of CTCs circulating in blood, we are currently investigating this using single-cell PCR and microarray analysis.

The most commonly used methods for detection of tumour cells in breast cancer are immunocytochemistry and molecular methods in the form of RT–PCR. There has been extensive research into methods for detection of occult micrometastases in patients before clinical manifestation. To date, bone marrow has been the most common site investigated for micrometastatic organ involvement. This is probably because of easy accessibility and the physiological absence of epithelial cells in the bone marrow, and also because the bone marrow is a homing site and blood is a transition compartment, two different biological compartments in a metastatic cascade (Pantel and Brakenhoff, 2004; Pantel *et al*, 2008).

We have monitored patients with primary breast cancer using sequential bone marrow aspirates for several years. Although these results indicate that a proportion of patients have PCR or immunocytochemical evidence of micrometastases, the test is confounded by the problem of sampling errors and in disease variability. In our study (Slade *et al*, 2005), we followed up 131 primary breast cancer patients for 4 years after surgery with bone marrow aspirates; 73% showed a fall in the micrometastatic load as measured by PCR and 63% as determined by ICC during follow-up. Of 91 patients who had repeat samples assayed, 87 and 65% had positive results at some time using PCR and ICC, respectively.

Patients are reluctant to have bone marrow tests more often than every 6 months, and thus it is difficult to even repeat the test to see whether the result is consistent. The CellSearch system, requiring only a peripheral blood sample, can be used frequently during follow-up. Our results indicate that either CTC sampling or a combination of blood and bone marrow tests may provide a practical monitoring system for breast cancer patients on follow-up. In our experience, many patients find repeat bone marrow sampling difficult and painful; this study suggests that the number of CTCs found on blood sampling is similar to the number found in bone marrow samples. Thus, blood tests may, in the future, be used in place of bone marrow sampling. However, before this can be recommended, we believe that a prospective study should be done comparing both tests.

Others have shown that the persistence of DTCs after treatment indicates a poor prognosis. Braun *et al* (1999, 2000a) investigated the effect of 500 mg of Edrecolomab on bone marrow micrometastases in 10 primary breast cancer patients before and at days 5–7 after antibody treatment. They showed a reduction in the number of disseminated cells after therapy. We (Smith *et al*, 2000) analysed 145 blood samples obtained from 22 metastatic breast cancer patients, both by immunocytochemistry and PCR, over 13 months. Of the 25 assessable courses of treatment, PCR agreed with the clinical outcome in 17 cases (68%) and ICC in 12 cases (48%). When 356 disease-free patients (Wiedswang *et al*, 2003) were subsequently analysed with a second bone marrow aspirate after a 3-year follow-up, the presence of micrometastases at this stage in disease-free patients was shown to be an independent prognostic factor (Wiedswang *et al*, 2004). Stathopoulou *et al* (2003) used real-time RT–PCR to study 77 patients with primary breast cancer before and after adjuvant chemotherapy, and showed a marked reduction both in the number of positive patients (31.2–6.5%) and in the level of positivity. They also studied 47 patients with overt metastases before and after chemotherapy and found no differences either in the number of patients positive (40.4 and 42.6%) or in the levels of positivity. In a study of 228 patients followed up with a repeat bone marrow aspirate 21 months after diagnosis, Janni *et al* (2005) showed that recurrence-free survival in patients with no DTCs was 149.7 months compared with 86.5 months in patients positive for DTCs (*P*=0.0003) and that overall survival was 162.1 months compared with 98.7 months (*P*=0.0008), respectively.

Recently, the need for a monitoring system has been highlighted by several trials indicating that sequential treatment during the disease-free period may improve overall survival in breast cancer. Thus, the Inter-Group Exemestane Study (IES) and Arimidex-Noluadex (ARNO)/Austrian Breast Cancer Study Group (ABCSG) studies both indicate that this approach may be preferable, as does the concept of sequencing chemotherapy, such as indicated by the studies using anthracyclines followed by taxanes. We feel that further adjuvant therapy during the follow-up period may yield better results, providing that these patients are selected on the basis of residual DTCs or CTCs.

## Figures and Tables

**Table 1:** Patient characteristics, details of therapy and timing of bone marrow and peripheral blood sampling in group II patients

**Patient no.**	**Date of diagnosis**	**Tumour type**	**Size (mm)**	**Grade**	**No. of LN**	**ER status**	**PR status**	**Therapy during study**	**Time from diagnosis BM1 (months)**	**QPCR**	**ICC**	**Time from diagnosis PB1 (months)**	**CTCs**	**Time from diagnosis PB2 (months)**	**CTCs**	**Time from diagnosis PB3 (months)**	**CTCs**	**Time from diagnosis PB4 (months)**	**CTCs**	**Time from diagnosis PB5 (months)**	**CTCs**	**Time from diagnosis PB6 (months)**	**CTCs**	**Time from diagnosis PB7 (months)**	**CTCs**
1	03/03/1998	IDC	20	2	0\15	2+	3+	Nil	89	0.046	0	94	0	100	0	110	0	114	0	118	1	121	0		
2	23/03/1998	IDC	9	2	LN-	3+	2+	Nil	96	0	0	96	ND	102	0										
3	19/05/1998	IDC	10	2	0\26	2+	3+	Nil	90	0	0	92	0	98	0	109	0	112	0	117	0				
4	26/05/1998	IDC	7	1	LN-	3+	Neg	Nil								108	0	111	0	114	0	117	0	120	0
5	21/07/1998	2 foci and tubulo-lobular	10	1	0\22	3+	3+	Nil	87	0	0	90	0	96	0	106	1	109	0		ND	114	1	118	0
6	03/11/1998	IDC	10	2	0\20	3+	3+	Nil								101	0	104	0	107	0	110	0	114	0
7	03/11/1998	IDC	17	1	0\15	3+	3+	Nil	92	0	0	92	0		ND										
8	25/05/1999	Multifocal ILC	10	2	0\34	Pos	Pos	Nil								100	1		ND	106	0				
9	06/06/2000	IDC	15	3	0\27	Neg	Neg	Nil								81	0	84	0	88	0	91	0	94	0
10	24/10/2000	IDC	10	1	0\27	3+	3+	Nil	62	0.032	0		ND	68	0	78	0	81	0		ND	87	2		
11	26/02/2001	ILC	18	2	0\29	3+	1+	Nil	52	0	0	60	0	66	0	76	0	79	0	82	0	85	1		
12	06/10/2003	IDC	1.5	3	0\4	Neg	—	Nil	17	0.002	0	29	0	35	0										
13	12/07/2002	IDC	12	3	0\17	Neg	Neg	Nil	33	0.023	0	43	0	49	0	59	0		ND	66	0	69	0		
14	10/12/2002	2 areas and IDC	20 and 11	1 and 2	0\28	3+	3+	Tam								54	0	57	0	60	0	63	0		
15	07/01/2003	IDC	19	3	0\31	3+	1+	Nil	29	0	1	36	0	42	0	53	1	56	2	59	0	63	0		
16	25/02/1994	IDC	17	3	LN-	Neg	Neg	Nil	135	0	0	135	ND	141	0	159	0	162	0		ND	169	1		
17	22/12/1997	IDC	17	3	0\16	2+	3+	Nil	90	0.006	0	96	0	102	0										
18	07/03/2000	IDC	10	2	0\23	3+	1+	Nil	60	0.018	0	68	0	74	0	88	0	91	0	94	0	97	0		

BM=bone marrow; CTCs=circulating tumour cells; DTCs=disseminated bone marrow tumour cells; ER=oestrogen receptor; ICC=immunocytochemistry; IDC=infiltrating ductal carcinoma; ILC=infiltrating lobular carcinoma; ND=not done; Neg=negative; PB=peripheral blood; Pos=positive; PR=progesterone receptor; QPCR=quantitative PCR; Tam=patient receiving tamoxifen at the time of analyses, all other patients were receiving no therapy. A CK-19 : ABL ratio of ⩾0.1 by RT–PCR is taken as positive.

Results for CTCs and DTCs in 18 group II T_1_N_0_ primary breast cancer patients.

**Table 2:** Patient characteristics, details of therapy and timing of bone marrow and peripheral blood sampling in group III patients

**Patient no.**	**Date of diagnosis**	**Tumour type**	**Size (mm)**	**Grade**	**No. of LN**	**ER status**	**PR status**	**Therapy during study**	**Time from diagnosis BM1 (months)**	**QPCR**	**ICC**	**Time from diagnosis BM2 (months)**	**QPCR**	**ICC**	**Time from diagnosis PB1 (months)**	**CTCs**	**Time from diagnosis PB2 (months)**	**CTCs**	**Time from diagnosis PB3 (months)**	**CTCs**	**Time from diagnosis PB4 (months)**	**CTCs**	**Time from diagnosis PB5 (months)**	**CTCs**	**Time from diagnosis PB6 (months)**	**CTCs**	**Time from diagnosis PB7 (months)**	**CTCs**
19	09/01/1999	IDC	30	3	4\13	3+	—	Arim	78	0.12	0	84	0.25	2	77	2		ND	99	0		ND	105	0	108	0	111	1
20	09/01/1995	IDC	30	3	12\17	1+	1+	Nil	126	0.18	1	132	0.11	1	125	0	132	0	149	0	152	1	156	2	159	0		
21	07/01/1994	ILC	>50	3	12\16	—	—	Nil	137	0.10	0	143	0.47	7	135	5	140	0	162	0	165	0	167	1	170	0		
22	05/01/1997	IDC	27	3	6\19	Neg	Neg	Nil	102	0.22	3	108	0.08	0	102	1	108	0	125	1	128	1		ND	134	0		
23	01/01/1996	IDC	33	3	4\20	—	—	Nil	117	0.03	0	123	0.00	0	119	0	125	0	138	0	141	1	144	0	147	0		
24	09/01/1996	ILC	55	2	5\29	3+	2+	Nil	129	0.02	1	135	0.10	1	119	0	124	0	138	0	142	0		ND	148	0		
25	23/12/1997	Tubular	11	-	2\13	Pos	Pos	Tam											113	0	117	0	121	0	123	0		
26	12/03/1997	IDC	16	2	16\36	1+	1+	Arim	92	0.14	0	104	0.00	0	98	0	100	0	122	0	126	0	129	0	132	0		
27	08/01/1999	IDC	10	2	5\15	3+	3+	Arim	68	0.22	1	74	0.04	0	76	0	82	0	100	0	104	0	107	0	112	0		
28	01/01/1997	IDC	20	3	8\21	Neg	Neg	Nil	107	0.20	3	113	0.20	0	106	0	112	2	Relapsed with metastatic disease 115 months after diagnosis									
29	11/01/1998	IDC	30	3	6\18	Neg	Neg	Nil	78	0.12	1	84	0.20	1	84	0	90	0										
30	02/01/2000	IDC	13	3	9\21	Pos	Neg	Arim	70	0.18	1	76	0.32	0		ND	76	0	89	0	92	0	94	1	97	0	100	0
31	05/01/1998	IDC	25	3	6\11	Pos	—	Nil	91	0.03	6	97	0.09	0	91	0		ND	113	0	116	0	119	0	121	0	124	1
32	03/01/1997	IDC	60	3	4\31	Neg	Neg	Nil	104	0.03	0	110	0.52	1	104	0	110	0	125	1	128	0	131	0	133	0		
33	27/09/1994	IDC	23	3	9\29	—	—	Nil											151	0	154	2	157	0	161	0	164	0
34	03/01/1995	Both IDC	2 × 16 and 22	3	9\24	1+	1+	Nil	126	0.06	0	132	0.13	3	132	8	136	0										
35	05/01/1997	IDC	50	3	4\22	Neg	Neg	Nil	95	0.02	0	101	0.00	0	95	0	101	0	125	0		ND		ND	132	0		
36	09/01/1997	T2 N1	25	2	11\20	2+	2+	Nil	90	0.06	0	96	0.06	1	96	0	109	0	124	0	127	0	130	1	133	1	136	0
37	10/01/1995	ILC	35	2	9\21	—	—	Nil	122	0.01	0	128	0.07	0	123	0	127	0	149	0	152	0		ND				
38	06/01/1999	IDC plus DCIS	25	3	4\29	2+	2+	Arim	78	0.01	0	84	0.05	0	78	0	84	0	100	1	103	1	107	23	112	0		
39	09/01/1997	IDC	40	3	10\21	1+	1+	Nil	93	0.02	1	99	0.00	0	99	0	103	0	126	1		ND	130	0	133	0		
40	02/01/1999	IDC	45	3	6\14	Pos	Pos	Arim	84	0	0	90	0.13	0	84	0	90	0	105	0	108	1	111	0				
41	03/01/1998	IDC	40	2	7\27	Pos	Pos	Arim	94	0.07	0	100	0.07	0	93	0	100	0	115	0	118	0	120	0				
42	08/12/1997	Multifocal IDC	>50	2	9\20	2+	3+	Arim											112	0	115	1	119	0	122	1	125	2
43	09/01/1997	TX N4	—	3	14\25	Neg	Neg	Nil	95	0.04	1	101	0.03	0	101	1	105	0										
44	04/01/1998	IDC	60	2	8\19	—	—	Tam	74	0.11	0	86	0.23	1		ND	96	7	Relapsed with metastatic disease 101 months after diagnosis									
45	05/09/1995	IDC	18	2	8\13	2+	Neg	Nil											144	0	147	0	151	0				
46	08/01/1997	IDC	20	2	7\19	3+	3+	Nil	85	0.12	1	97	0.26	0	101	0	106	0	126	0	129	1	132	0	135	0		
47	29/12/2003	IDC	15	3	45\45	Neg	Neg	CT (FEC)											41	0	44	0		ND	49	0	53	0
48	16/12/1998	Mucoid carcinoma	50	1	8\19	2+	3+	Arim	72	0.03	0	84	0.05	0	84	3	97	0	103	0	106	0	109	1	112	0		
49	08/01/1999	IDC	20	3	4\26	Neg	Neg	Nil	61	0.33	2	72	0.53	0	77	1	83	0	99	1		ND	105	0	108	0	111	0
50	02/01/2001	IDC	14	2	20\39	3+	3+	Arim	49	0.00	0		ND	ND	61	0	65	0	77	1	81	1	84	0	88	1		
51	01/01/2000	Multifocal lobular carcinoma	17	3	6\18	3+	3+	Arim	37	0.08	1	51	0.03	0	47	0	51	0	87	0	90	0	93	0	97	0	100	1

Arim=arimidex; BM=bone marrow; CT=chemotherapy; CTCs=circulating tumour cells; DCIS=ductal carcinoma *in situ*; DTCs=disseminated bone marrow tumour cells; ER=oestrogen receptor; FEC=fluorouracil, epirubicin and cyclophosphamide; ICC=immunocytochemistry; IDC=infiltrating ductal carcinoma; ILC=infiltrating lobular carcinoma; ND=not done; Neg=negative; PB=peripheral blood; Pos=positive; PR=progesterone receptor; QPCR=quantitative PCR; Tam=tamoxifen.

High-risk patients therapy and monitoring details.

Results for CTCs and DTCs in 33 group III high-risk primary breast cancer patients.
